# A raw data article on the physico-chemical properties of soil from six firkas in Dharmapuri district, Tamil Nadu, India

**DOI:** 10.1016/j.dib.2022.108452

**Published:** 2022-07-09

**Authors:** M. Ramya, S. Sathiyamurthi

**Affiliations:** Department of Soil Science and Agricultural Chemistry, Faculty of Agriculture, Annamalai University, Annamalai Nagar-608002, India

**Keywords:** Soil properties, Physico, Chemical properties, Spatial variability, Dharmapuri district

## Abstract

The present study was aimed to assess the physical and chemical characteristics of soil belonging to six firkas covering three blocks of Dharmapuri district. A total of 125 samples were collected by means of Global Positioning System (GPS). The processed soil samples were analyzed for pH, Electrical conductivity (EC), soil separates, Bulk density (BD), Water holding capacity (WHC), organic carbon (OC), calcium carbonate (CaCO_3_), available nitrogen (AVN), available phosphorus (AVP), and available potassium (AVK). Extreme value in the data set was removed by outlier removal algorithm. Spatial variability maps were prepared using the kriging method using ArcGIS 10.4 . The best fitted semi variance model for pH, EC, AVK and CaCO_3_ was spherical; BD, sand, silt, and OC was circular; a Gaussian model was best fitted for WHC and AVN, while clay and AVP were exponential. The data presented in this study will help farmers, land managers, and policymakers to mitigate land degradation, and other environmental issues, thereby helping to increase land productivity.

## Specification Table

**Table:** 

Subject area	Agriculture and Biological science
Specific subject area	Soil Science
Type of data	Tables and Images
How the data were acquired	The study area boundary was extracted using SOI Toposheet (57H/14, 57H/15, 57L/3, and 57L/4) from GSI, a cadastral map, and Block boundary map from local authorities. The soil sample's location was determined by the regular point sampling method. The soil samples were collected during fallow period (February and April, 2021). The soil sample's location was recorded using GARMIN 76 CS x GPS device.
Data format	Raw and Analyzed data
Description of data collection	The soil sample's location was predetermined by regular point generation technique and recorded using a GPS device. The surface litters and grass were removed and ‘v’ shaped pit was dug at the depth of 15 cm. After cleaning the pit, one-inch thickness soil was collected from each side. Using a wooden mallet, the air-dried soil samples were ground into powder. The samples were mixed and reduced in volume to roughly 500 g by quartering method. The samples were stored in air tight container and analyzed for pH, EC, particle size analysis, BD, WHC, OC, CaCO_3_ and available NPK by following the standard procedures. These data were converted into vector layers and kriging was applied to create a spatial variable map for each soil parameters.
Data source location	The study area is the part of Dharmapuri district,Tamilnadu, India. Latitude ranges between 11⁰ 45’ and 12⁰ 15’ N and longitude ranges between 77⁰ 30’ and 78⁰ 30’E, encompassing about 482 sq.km. The elevation of the study area ranged from 403 to 1213 m MSL.
Data accessibility	Data accessibility Repository name: Mendeley Data, Title of the data set: A raw data article on the physical and chemical properties of soil from six firkas in Dharmapuri district, Tamil Nadu, India. Data identification number: 10.17632/nm5b82wbmt.4. Direct URL to data: https://data.mendeley.com/datasets/nm5b82wbmt/3

## Value of the Data

• This data set will be used to determine the agricultural land suitability and crop suitability of the study area.
• This data will help the land manager and policymakers to suggest an action plan for sustainable crop production.
• Furthermore, this data will aid other science and engineering fields in better understanding the soil in the study region, and it can be used for educational and research purposes.

## 1 Data Description

**Fig. 1:**
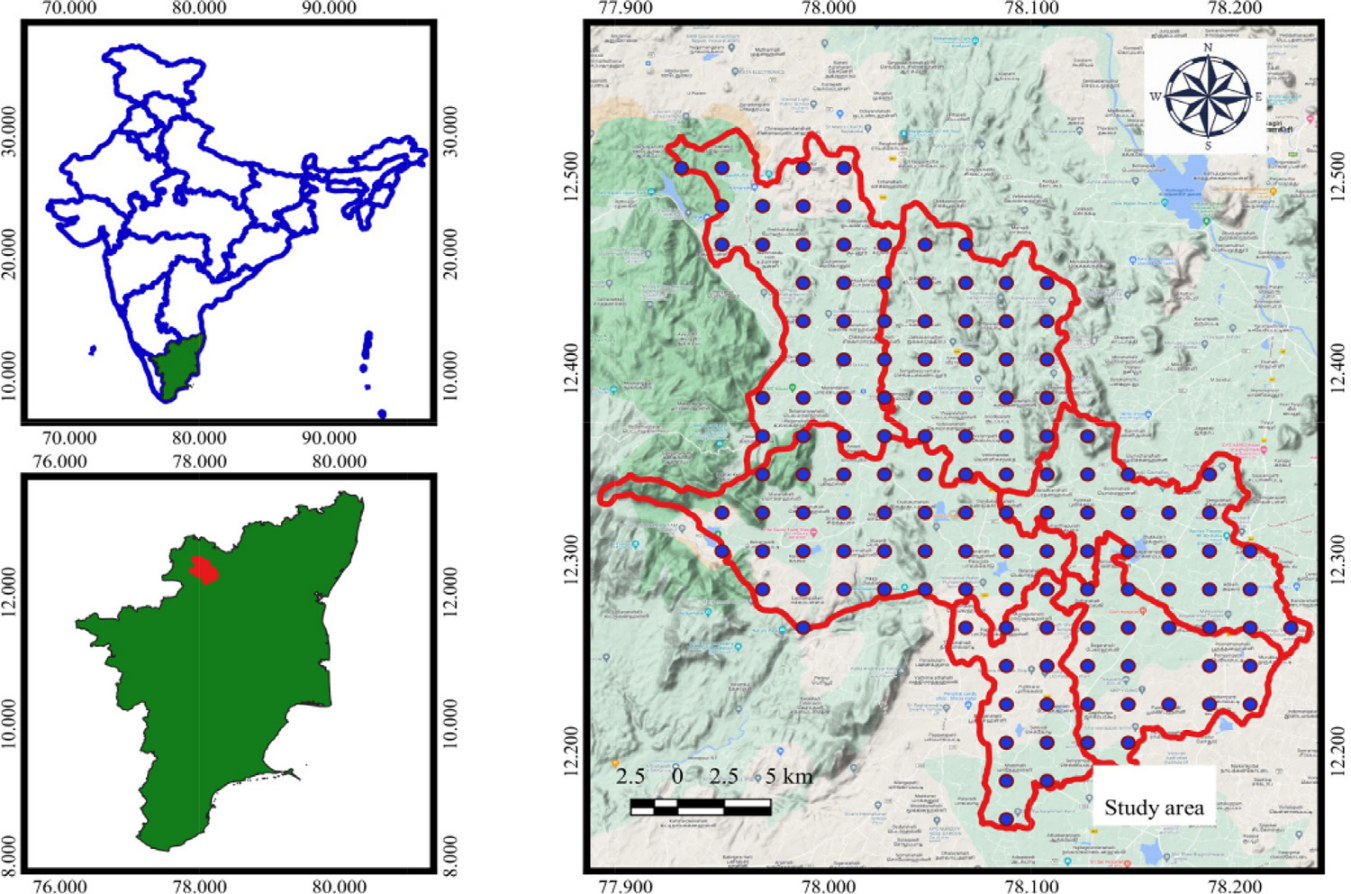
Study area and soil sample's locations

**Fig. 2:**
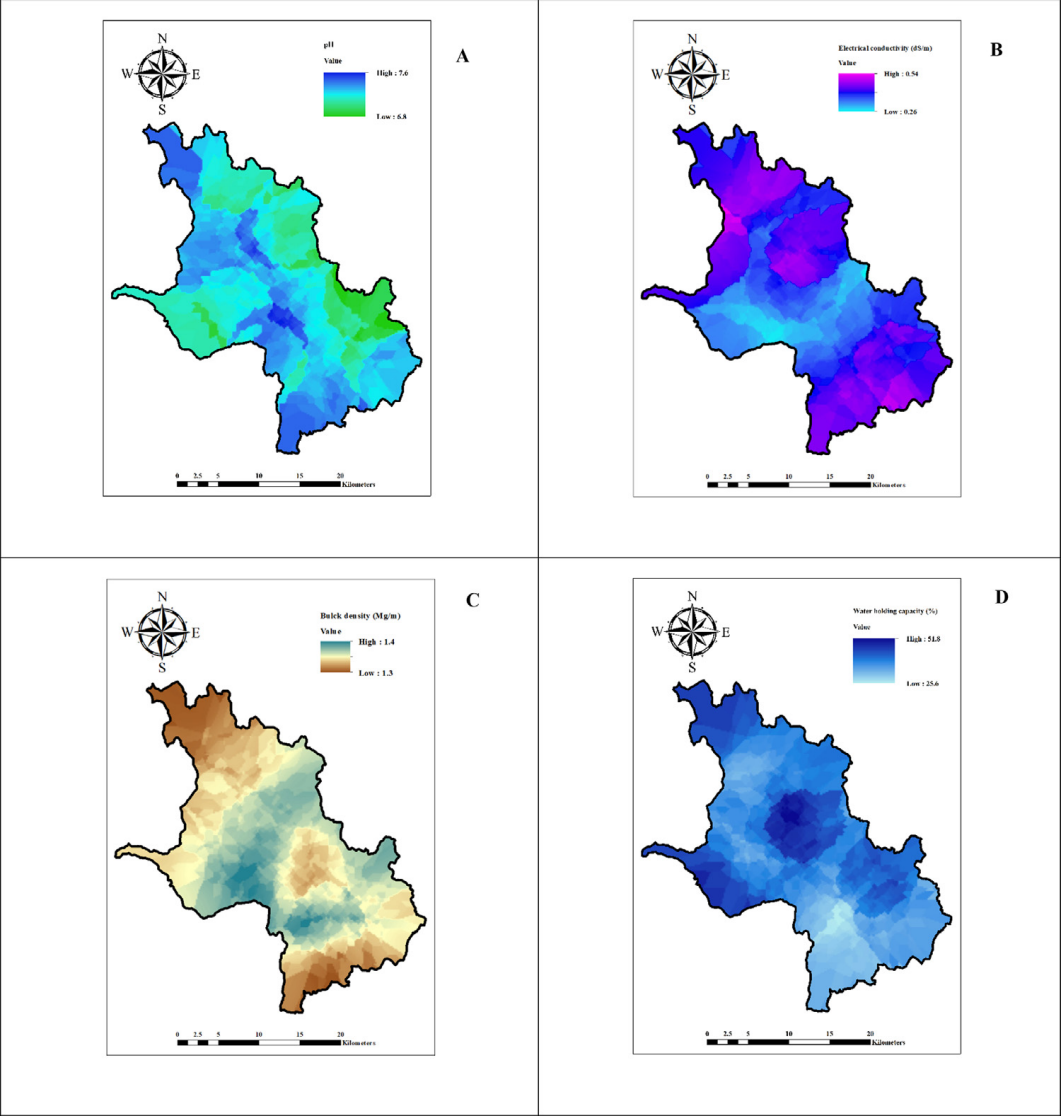
Spatial distribution maps of (A) Soil pH, (B) Soil EC, (C) Bulk density, (D) Water holding capacity

**Fig. 3:**
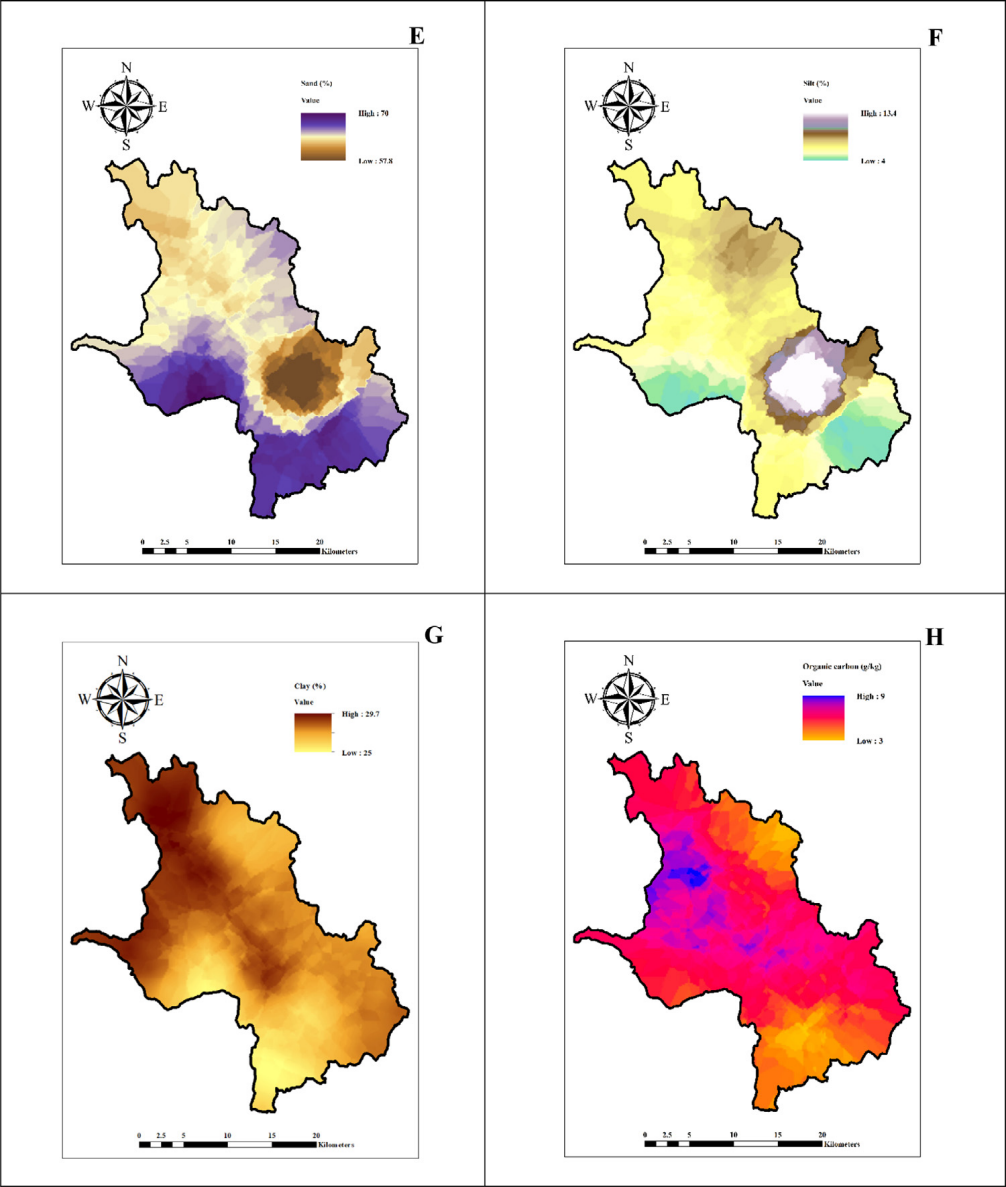
Spatial distribution maps of (E) Sand content, (F) Silt content, (G) Clay content, (H) Organic carbon.

**Fig. 4:**
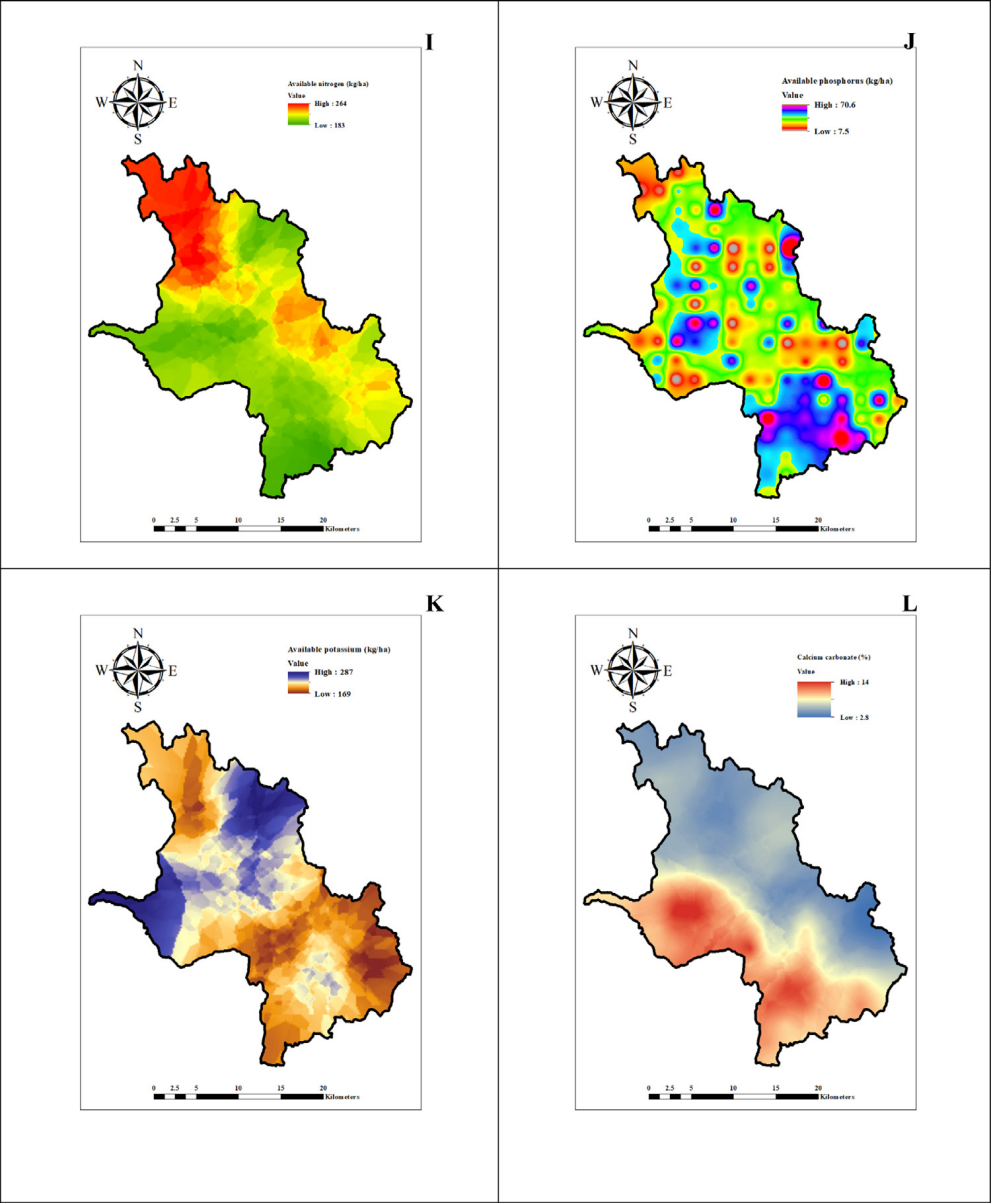
Spatial distribution maps of (I) Available Nitrogen, (J) Available Phosphorus, (K) Available Potassium, (L) Calcium Carbonate.

The data represents the physico-chemical properties of soil located in six firkas of Dharmapuri district. Table 1, Table 2, Table 3, Table 4, Table 5, Table 6 presents the physical and chemical properties of the soil in the study area. Table 7 presents the descriptive statistics of the soil properties. Fig. 1 shows the study area map and sample's location. Fig. 2, Fig. 3, Fig. 4 depict thematic maps showing the physical and chemical parameters of soil. Fig. 2 depicts information on the pH, EC (dS/m), BD (Mg/m^3^), WHC (%), where Fig. 3 represents sand (%), silt (%), clay (%) and OC (g/kg), and Figure 4 reveals thematic map of the available NPK, (kg/ha), and CaCO_3_ (%).

**Table 1:** The Physico-chemical properties of the Marandahalli firka soil samples from the Dharmapuri district in Tamil Nadu, India.

Sample no	Latitude	Longitude	pH	EC (dS/m)	Sand (%)	Silt (%)	Clay (%)	Textural class	BD (Mg/m^3^)	WHC (%)	OC (g/kg)	CaCO_3_ (%)	AVN (kg/ha)	AVP (kg/ha)	AVK (kg/ha)
**M1**	12.5049	77.98748	7.5	0.3	67.80	4.5	27.7	SCL	1.3	47	5.2	3	252	19	183
**M2**	12.50488	78.00748	7.8	0.4	65.80	5.0	29.2	SCL	1.2	60	4.2	5	248	21	196
**M3**	12.48491	77.94751	7.7	0.2	65.80	7.0	27.2	SCL	1.2	46	5.8	4.5	279	19	210
**M4**	12.48499	77.9675	7.8	0.3	61.30	8.0	30.7	SCL	1.3	64	4	3.5	263	29	199
**M5**	12.48485	77.98753	7.5	0.3	64.55	6.25	29.2	SCL	1.2	46	7.3	5.5	250	36	171
**M6**	12.4848	78.00751	7.6	0.3	61.30	8.0	30.7	SCL	1.3	44	7.3	7	248	34	160
**M7**	12.46486	77.94742	7.7	0.4	65.80	7.0	27.2	SCL	1.4	54	9.1	5.5	246	20	328
**M8**	12.46477	77.9675	7.9	0.3	63.30	5.0	31.7	SCL	1.2	34	0.6	8.5	176	27	210
**M9**	12.46501	77.98753	7.3	0.4	64.99	5.3	29.71	SCL	1.3	42	6.9	5.6	253	36	195
**M10**	12.46491	78.00751	7.5	0.88	63.30	4.5	32.2	SCL	1.3	40	5.2	7	320	21	123
**M11**	12.46501	78.02749	6.4	0.25	68.80	5.5	25.7	SCL	1.5	34	7.4	1.5	267	61	279
**M12**	12.4483	77.98734	7.2	0.52	65.48	5.6	28.92	SCL	1.3	32	6.9	5.2	262	37	153
**M13**	12.44507	78.00756	7.3	0.82	65.80	7.0	27.2	SCL	1.5	30	3.6	5.5	289	34	65
**M14**	12.44488	78.02753	7	0.53	65.30	7.0	27.7	SCL	1.3	31	5.2	5	263	20	169
**M15**	12.42491	77.98747	7.4	0.38	60.30	9.5	30.2	SCL	1.3	39	10.3	7	229	34	180
**M16**	12.42472	78.0074	7.7	0.38	65.80	4.5	29.7	SCL	1.2	44	10.6	2	332	45	209
**M17**	12.40508	77.98756	7.2	0.38	67.80	4.5	27.7	SCL	1.3	32	11.7	5.5	229	36	180
**M18**	12.40498	78.0075	7	0.34	65.65	4.5	29.85	SCL	1.3	24	9.7	4.6	246	41	199
**M19**	12.40493	78.02752	7.8	0.37	63.30	4.5	32.2	SCL	1.4	22	4.5	8	248	29	81
**M20**	12.38503	77.96751	7.1	1.3	66.05	6.25	27.7	SCL	1.3	39	6.5	4.5	263	34	225
**M21**	12.38499	77.98754	7.4	0.42	64.63	7.0	28.37	SCL	1.5	30	2.7	5	238	45	254
**M22**	12.38503	78.00752	7.9	0.32	65.80	5.0	29.2	SCL	1.4	32	9.5	7	248	54	328
**M23**	12.38484	78.02746	7.7	0.27	67.80	4.5	27.7	SCL	1.4	60	5.8	1	307	36	210
**M24**	12.36491	77.96754	7.4	0.3	61.30	8.0	30.7	SCL	1.3	64	3.7	5.5	248	17	180
**M25**	12.36493	78.0075	7.5	0.47	70.30	2.0	27.7	SCL	1.3	18	12.9	8.5	116	53	424
**M26**	12.3649	78.02753	7.8	0.32	63.80	7.0	29.2	SCL	1.3	47	1.2	3	232	34	488

*EC – Electrical conductivity, BD - Buk density, WHC – Water holding capacity, OC – Organic carbon, AVN-Available nitrogen, AVP-Available phosphorus, AVK-Available potassium

**Table 2:** The Physico-chemical properties of the Vellichandai firka soil samples from the Dharmapuri district in Tamil Nadu, India.

Sample no	Latitude	Longitude	pH	EC (dS/m)	Sand (%)	Silt (%)	Clay (%)	Textural class	BD (Mg/m^3^)	WHC (%)	OC (g/kg)	CaCO_3_ (%)	AVN (kg/ha)	AVP (kg/ha)	AVK (kg/ha)
**V27**	12.4649	78.04752	7.7	0.5	65.8	7	27.2	SCL	1.3	45	1.5	4.5	220	28	328
**V28**	12.44497	78.04755	7.5	0.36	69.3	8	25.95	SCL	1.2	30	0.3	5.2	130	28	378
**V29**	12.44492	78.06743	7.3	0.43	67.8	8	25.82	SCL	1.3	38	1.8	4	153	28	355
**V30**	12.42493	78.02753	7.4	0.37	60.3	9.5	30.2	SCL	1.4	25	12.4	3	263	56	403
**V31**	12.42487	78.04752	7.6	0.25	68.3	7	24.7	SCL	1.3	42	0.2	2	144	3	333
**V32**	12.42499	78.06753	7.2	0.57	66.3	8	25.7	SCL	1.4	46	4.6	8	176	29	332
**V33**	12.42497	78.08753	7.4	0.33	68.3	4.5	27.2	SCL	1.4	38	6.2	7	220	50	326
**V34**	12.42492	78.10754	7.2	0.48	66.05	6.25	27.7	SCL	1.4	39	3.1	5.5	279	77	452
**V35**	12.40484	78.04752	6.7	0.3	60.3	9.5	30.2	SCL	1.3	39	8.8	2.5	279	51	517
**V36**	12.40494	78.0675	8	0.32	61.3	9.5	29.2	SCL	1.4	64	4.6	4.5	191	35	352
**V37**	12.40494	78.08753	6.3	0.29	68.3	4.5	27.2	SCL	1.5	28	4.6	7	204	9	196
**V38**	12.40494	78.10756	6.6	0.24	63.8	8	28.2	SCL	1.5	37	5.8	8	232	44	112
**V39**	12.38496	78.04752	7.3	0.37	64.63	7	28.37	SCL	1.4	47	12	8	198	27	303
**V40**	12.38496	78.06746	6.6	1.24	65.8	7	27.2	SCL	1.4	26	4	7	260	53	186
**V41**	12.38496	78.08748	7.9	0.48	68.3	7	24.7	SCL	1.4	33	3.9	5.5	326	29	338
**V42**	12.38496	78.10747	6.9	0.34	66.3	5.5	28.2	SCL	1.4	38	3.7	3.5	229	22	225
**V43**	12.3649	78.04755	7.3	0.43	65.6	6	28.4	SCL	1.5	70	0.6	5	116	21	119
**V44**	12.36495	78.06753	7.9	0.43	65.8	5	29.21	SCL	1.4	108	11.1	3.5	238	38	198
**V45**	12.36488	78.08751	7.4	0.3	63.3	5	31.7	SCL	1.3	33	2.7	9.5	201	21	372
**V46**	12.36488	78.10754	7	0.27	66.8	5.6	27.6	SCL	1.3	32	3.6	6.5	219	26	252
**V47**	12.34478	78.06753	6.9	0.65	61.3	8	30.7	SCL	1.4	37	4	5	276	22	338
**V48**	12.34497	78.08751	7.4	0.2	70.8	6.5	22.7	SCL	1.4	32	8.9	2.5	194	25	350

*EC – Electrical conductivity, BD - Buk density, WHC – Water holding capacity, OC – Organic carbon, AVN-Available nitrogen, AVP-Available phosphorus, AVK-Available potassium

**Table 3:** The Physico-chemical properties of the Palacode firka soil samples from the Dharmapuri district in Tamil Nadu, India.

Sample no	Latitude	Longitude	pH	EC (dS/m)	Sand (%)	Silt (%)	Clay (%)	Textural class	BD (Mg/m^3^)	WHC (%)	OC (g/kg)	CaCO_3_ (%)	AVN (kg/ha)	AVP (kg/ha)	AVK (kg/ha)
**Pal49**	12.34495	78.00751	7.5	0.37	63.8	7	29.2	SCL	1.3	32	7.1	9	138	58	220
**Pal50**	12.34502	78.02752	7.3	0.34	63.8	7	29.2	SCL	1.4	39	8	14.5	194	52	192
**Pal51**	12.3449	78.04752	7.6	0.57	68.3	5	26.7	SCL	1.4	53	11.7	5.5	182	29	163
**Pal52**	12.32508	77.94757	7.4	0.32	63.3	5	31.7	SCL	1.2	61	4.9	13	235	14	517
**Pal53**	12.32504	77.96755	7.7	0.25	68.8	4.5	26.7	SCL	1.5	29	8.3	8.5	160	10	251
**Pal54**	12.32494	77.98753	7.4	0.39	64.6	6.6	28.8	SCL	1.3	22	9.5	21.5	201	57	235
**Pal55**	12.32499	78.00742	7.5	0.31	67.02	6.15	26.83	SCL	1.4	26	7.1	17	198	46	188
**Pal56**	12.32484	78.02759	6	0.2	70.8	6.5	22.7	SCL	1.4	24	8.8	13	226	36	132
**Pal57**	12.32489	78.04752	7.8	0.33	67.6	4.57	27.83	SCL	1.5	37	7.4	14	176	16	161
**Pal58**	12.32513	78.06764	7.9	0.31	63.63	6.64	29.74	SCL	1.5	36	6.3	8.5	211	26	287
**Pal59**	12.30515	77.9676	7.3	0.36	63.3	5	31.7	SCL	1.4	59	7	5	213	40	517
**Pal60**	12.30491	77.98753	7.6	0.29	63.3	7	29.7	SCL	1.4	56	8.3	15	201	13	191
**Pal61**	12.30505	78.00742	7	0.25	68.8	4.5	26.7	SCL	1.5	23	3.4	9.5	226	33	165
**Pal62**	12.30496	78.02749	7.3	0.28	70.3	2	27.7	SCL	1.5	31	4	9.7	254	33	273
**Pal63**	12.30505	78.04757	7.6	0.32	71.3	4.5	24.2	SCL	1.4	47	8.9	3.5	263	50	517
**Pal64**	12.30486	78.06741	7.6	0.37	61.3	7	31.7	SCL	1.3	30	6.6	11	166	25	448
**Pal65**	12.30496	78.08748	7.5	0.25	65.3	4.5	30.2	SCL	1.4	38	6.5	4.5	232	26	84
**Pal66**	12.30491	78.10751	7.4	0.29	58	13	29	SL	1.2	30	3.7	3	219	20	173
**Pal67**	12.28512	77.9675	6.1	0.4	68.55	3.5	27.95	SCL	1.3	72	4	8.5	210	39	280
**Pal68**	12.28493	77.98743	7.3	0.3	73.8	2	24.2	SCL	1.3	43	3	8.5	202	39	240
**Pal69**	12.28498	78.00751	7.5	0.2	72.8	2	25.2	SCL	1.4	63	1.5	12.5	194	25	172
**Pal70**	12.28498	78.02744	7.5	0.26	72.5	2.8	24.7	SCL	1.5	44	2.8	12.8	223	28	145
**Pal71**	12.28498	78.04752	7.7	0.3	73.8	4.5	21.7	SCL	1.5	38	3.7	13.5	223	26	118
**Pal72**	12.28517	78.06764	8.3	0.27	65.8	7.2	27	SCL	1.2	70	7.7	30	202	13	138
**Pal73**	12.28498	78.08748	7.4	0.38	65.3	4.5	30.2	SCL	1.4	12	4	2	201	17	125
**Pal74**	12.2649	77.98751	7	0.6	68.05	4.5	27.5	SCL	1.3	60	4	13.6	197	19	181

*EC – Electrical conductivity, BD - Buk density, WHC – Water holding capacity, OC – Organic carbon, AVN-Available nitrogen, AVP-Available phosphorus, AVK-Available potassium

**Table 4:** The physico-chemical properties of the Karimangalam firka soil samples from the Dharmapuri district in Tamil Nadu, India.

Sample no	Latitude	Longitude	pH	EC (dS/m)	Sand (%)	Silt (%)	Clay (%)	Textural class	BD (Mg/m^3^)	WHC (%)	OC (g/kg)	CaCO_3_ (%)	AVN (kg/ha)	AVP (kg/ha)	AVK (kg/ha)
**K75**	12.3449	78.10747	7.6	0.3	65.8	4.5	29.7	SCL	1.3	38	8	1.5	295	50	286
**K76**	12.3448	78.1275	7.3	0.34	66	6	28	SCL	1.3	35	12	1.5	326	22	276
**K77**	12.3449	78.1475	7.4	0.28	65.3	7	27.7	SCL	1.5	32	8.3	4	257	53	117
**K78**	12.34504	78.1875	6	0.14	66.3	8	25.7	SCL	1.5	54	3.4	4.5	157	38	88
**K79**	12.3249	78.0875	7.3	0.28	64.3	7	28.7	SCL	1.3	43	5.1	4.6	226	44	201
**K80**	12.3249	78.1075	7.3	0.29	63.3	9.5	27.2	SCL	1.3	58	11	4	254	15	194
**K81**	12.3248	78.1275	7.2	0.32	65.8	7	27.2	SCL	1.3	42	2.5	10.5	263	15	237
**K82**	12.3249	78.1475	7.1	0.25	68.3	4.5	27.2	SCL	1.5	41	1.5	3.5	229	13	307
**K83**	12.3248	78.1674	6	0.2	68.3	4.5	27.2	SCL	1.5	22	3.4	5.5	254	11	80
**K84**	12.3249	78.1874	7.5	0.71	63.3	7	29.7	SCL	1.4	37	3.4	1	188	48	261
**K85**	12.305	78.1274	7.7	0.49	43.9	28.15	27.95	CL	1.3	33	12	15.5	245	12	174
**K86**	12.3051	78.1675	7	0.33	68.3	4.5	27.2	SCL	1.3	40	10.6	4	304	10	229
**K87**	12.30478	78.18751	7.9	0.5	65.8	6.5	27.7	SCL	1.3	45	7	2.5	151	28	186
**K88**	12.3049	78.2075	7.8	0.56	65.8	7	27.2	SCL	1.3	35	13.6	1.5	279	27	168
**K89**	12.2849	78.1674	7.4	0.37	65.3	4.5	30.2	SCL	1.3	26	6.1	5	229	31	133
**K90**	12.2849	78.1874	7.6	0.34	67.8	4.5	27.7	SCL	1.5	27	5.5	6.5	215	28	151
**K91**	12.2849	78.2074	7.6	0.29	63.8	5	26.2	SCL	1.4	39	3.1	3	129	30	151
**K92**	12.2649	78.1874	6	0.26	65.8	4.5	29.7	SCL	1.3	15	8.2	4.5	241	25	201
**K93**	12.2649	78.20747	7.2	0.33	66	6.4	27.7	SCL	1.4	32	4	6	254	58	251
**K94**	12.26491	78.2275	7.5	0.26	65.3	4.5	30.2	SCL	1.3	23	3.9	6.5	235	17	257

*EC – Electrical conductivity, BD - Buk density, WHC – Water holding capacity, OC – Organic carbon, AVN-Available nitrogen, AVP-Available phosphorus, AVK-Available potassium

**Table 5:** The physico-chemical properties of the Pulikarai firka soil samples from the Dharmapuri district in Tamil Nadu, Indi

Sample no	Latitude	Longitude	pH	EC (dS/m)	Sand (%)	Silt (%)	Clay (%)	Textural class	BD (Mg/m^3^)	WHC (%)	OC (g/kg)	CaCO_3_(%)	AVN (kg/ha)	AVP (kg/ha)	AVK (kg/ha)
**Pul95**	12.28488	78.1075	7.3	0.32	55	15.75	29.25	SCL	1.3	19	11.4	6	174	47	272
**Pul96**	12.2649	78.0674	6.4	0.22	70.8	2	27.2	SCL	1.5	30	8	3	220	38	316
**Pul97**	12.2649	78.0875	7.5	0.32	68.8	3.3	27.9	SCL	1.4	27	2.8	7	211	22	153
**Pul98**	12.2649	78.1075	7.4	0.35	64.12	8.5	27.38	SCL	1.4	23	2.7	8.6	192	41	241
**Pul99**	12.2449	78.0875	7.5	0.6	73.3	3.5	23.2	SCL	1.5	40	2.5	12	213	62	399
**Pul100**	12.2448	78.1074	7.7	0.5	65.8	7	27.2	SCL	1.4	19	7.1	17	202	47	230
**Pul101**	12.225	78.0875	7.3	0.4	68.3	5.5	26.2	SCL	1.4	38	4.9	17	202	50	271
**Pul102**	12.2249	78.1074	7.3	0.46	63.3	7.5	29.2	SCL	1.4	37	5.2	11	191	39	144
**Pul103**	12.2049	78.0874	7.2	0.37	70.8	6	23.2	SCL	1.2	31	3.2	9	194	35	283
**Pul104**	12.2049	78.1074	7.62	0.54	70.8	6	23.2	SCL	1.2	27	3.4	4	166	32	447
**Pul105**	12.185	78.0875	7.9	0.33	68.3	6.4	25.3	SCL	1.3	36	6.8	15	223	40	119
**Pul106**	12.1849	78.1075	7.8	0.37	70.8	6	23.2	SCL	1.4	28	5.8	8.5	166	26	283
**Pul107**	12.1649	78.0875	7.8	0.59	63.3	7	29.7	SCL	1.3	36	7.4	7	191	33	180

*EC – Electrical conductivity, BD - Buk density, WHC – Water holding capacity, OC – Organic carbon, AVN-Available nitrogen, AVP-Available phosphorus, AVK-Available potassium

**Table 6:** The Physico-chemical properties of the Periyanahalli firka soil samples from the Dharmapuri district in Tamil Nadu, India.

Sample no	Latitude	Longitude	pH	EC (dS/m)	Sand (%)	Silt (%)	Clay (%)	Textural class	BD (Mg/m^3^)	WHC (%)	OC (g/kg)	CaCO_3_ (%)	AVN (kg/ha)	AVP (kg/ha)	AVP (kg/ha)
**Pe 108**	12.3048	78.1475	7.2	0.58	41.5	29.3	29.2	CL	1.3	86	5.2	4.1	251	26	250
**Pe 109**	12.2849	78.1275	7.1	0.29	46.3	27	26.7	SCL	1.4	26	1.8	13	147	47	419
**Pe 110**	12.2849	78.1475	7.4	0.33	70.3	4.5	25.2	SCL	1.4	59	8	5	226	69	291
**Pe111**	12.2649	78.1274	7.1	0.27	72.8	4.5	22.7	SCL	1.7	26	2.5	13	193	48	310
**Pe112**	12.2649	78.1475	7.8	0.44	68.3	4.5	27.2	SCL	1.3	29	4.2	4	241	21	288
**Pe113**	12.2649	78.1675	6.7	0.94	71.3	2	26.7	SCL	1.4	82	3.8	7	232	51	192
**Pe114**	12.2449	78.1275	7.2	0.43	69.3	5.75	24.95	SCL	1.5	23	4.6	15	197	49	290
**Pe115**	12.2448	78.1475	7	0.67	68.8	5.2	26	SCL	1.4	30	2.5	9	206	42	233
**Pe116**	12.2449	78.1675	7.9	0.31	69.72	3.51	26.77	SCL	1.4	31	1.8	10.5	182	56	123
**Pe117**	12.2448	78.1874	7.6	0.23	68.3	4.5	27.2	SCL	1.4	19	5.2	14	243	22	135
**Pe118**	12.2449	78.2075	8.2	0.38	68.3	4.5	27.2	SCL	1.2	44	2.5	7	292	14	420
**Pe119**	12.2249	78.1275	7.8	0.29	66.14	5.91	27.95	SCL	1.1	29	3	15.5	215	47	203
**Pe120**	12.225	78.1474	7.5	0.46	68.58	4.37	27.05	SCL	1.2	32	1.8	9.5	194	50	336
**Pe121**	12.2249	78.1675	7.9	0.42	70.8	2	27.2	SCL	1.2	39	8.6	4	163	63	517
**Pe122**	12.2249	78.1874	7.2	0.39	65.8	4.5	29.7	SCL	1.5	27	7.4	17	257	53	177
**Pe123**	12.2249	78.2074	7.2	0.87	67.05	4.5	28.45	SCL	1.3	36	5.5	8	135	24	149
**Pe124**	12.205	78.1274	7.4	0.53	68.47	5.95	25.58	SCL	1.3	36	0.6	9.7	141	39	325
**Pe125**	12.2049	78.1475	7.4	0.49	68.5	5.2	26.3	SCL	1.2	34	2.5	9.6	167	44	330

*EC – Electrical conductivity, BD - Buk density, WHC – Water holding capacity, OC – Organic carbon, AVN-Available nitrogen, AVP-Available phosphorus, AVK-Available potassium

**Table 7:** Descriptive statistics of the soil sample parameters of the six firkas in the Dharmapuri district of Tamil Nadu, India.

	Minimum	Maximum	Mean	Std. Dev.	CV	Skewness	Kurtosis
**pH**	6	8.3	7.4	0.44	0.04	-1.20	2.01
**EC (dS/m)**	0.14	1.3	0.34	0.18	0.02	2.52	8.62
**Sand (%)**	41.5	73.8	65.8	4.75	0.52	-2.41	10.01
**Silt (%)**	2	29.3	5.75	3.98	0.44	4.09	20.35
**Clay (%)**	21.7	32.2	27.7	2.17	0.24	-0.36	0.17
**BD (Mg/m^3^)**	1.1	1.7	1.4	0.09	0.01	0.18	0.14
**WHC (%)**	12	108	36	14.97	1.66	1.51	3.65
**OC (g/kg)**	0.2	13.6	5.2	3.12	0.34	0.51	-0.42
**CaCO_3_ (%)**	1	30	6.5	4.69	0.52	1.48	3.59
**AVN (kg/ha)**	116	332	223	45.39	5.04	-0.007	-0.05
**AVP (kg/ha)**	3	77	33	14.60	1.62	0.35	-0.38
**AVK (kg/ha)**	65	517	225	106.03	11.78	0.81	0.20

*EC – Electrical conductivity, BD - Buk density, WHC – Water holding capacity, OC – Organic carbon, AVN-Available nitrogen, AVP-Available phosphorus, AVK-Available potassium

## 2 Data, Experimental Design, Materials and Methods

### 2.1 Data

➢ The physical and chemical characteristics of surface soil samples including pH, EC (dS/m), sand (%), silt (%), clay (%), BD (Mg/m^3^), and WHC (%), OC (g/kg), CaCO_3_ (%) and available NPK (kg/ha) in the six selected firkas of Dharmapuri district were analyzed.
➢ The sampling locations are illustrated in Fig. 1 and the detailed soil characteristics are presented in Table 1–6.
➢ The data presented in the tables were based on the laboratory investigation.

## 3 Experimental Design

### 3.1 Study area description

The study area comprises six firkas namely Marandahalli, Vellichandai, Palacode, Karimangalam, Pulikarai, and Periyanahalli are located in the Dharmapuri district (Fig. 1), which covers latitudes between 11˚45’ and 12˚15’N and longitudes between 77˚30’ and 78˚30’E, encompassing approximately 482 km^2^ [1]. Regarding the climate, the hottest period of the study area was March to May, with maximum temperature of 38˚C and minimum temperature is about 17˚C which was recorded in January. The mean annual rainfall ranged from 900 to 1200 mm, through southwest and northeast monsoons. The study area is occupied by diverse range of igneous and metamorphic rocks.

### 3.2 Soil sample collection and laboratory analysis

The soil samples were collected between February and April, 2021. A Garmin 76 CSx Global Positioning Systems (GPS) device was used to record the exact soil sample's locations. The soil samples were taken from the plough layer after the surface litter was removed; a total of 125 samples were collected. At each sampling point, a 15-cm deep ‘v’-shaped cut was made using a spade, soil was collected from each side. The soil samples were air-dried and pulverized using a wooden mallet. The samples were sieved using a 2-mm sieve and packed in plastic bags or air tight containers. Furthermore, the soil samples were analyzed for pH [2], electrical conductivity [2], soil separates [3], bulk density [2], water-holding capacity [4], organic carbon [5], calcium carbonate [6], available N [7], available P [8], and available K [9], and the data analyzed statistically to remove outlier values. The analyzed data are presented in the table format.

## 4 Spatial Variability Map Preparation

A spatial variability map of the study area was generated using the kriging method in the ArcGIS 10.4 software [10]. The kriging technique was used to underestimate the lower values and exaggerate larger values by applying a smooth model of spatial variability to the dataset and limiting its fitting errors. Using the kriging method, a smooth surface was created by reducing the errors generated by variance in the fit of the model to each neighborhood. The kriged surface was generalized for all the parameters by sub-setting the mean of the surrounding pixels and substituted in the sampled point. The variance error was used to detect issues in the sample point, model parameters, and local neighborhood design. Before using the ordinary kriging method, the semi variance was calculated to identify the interpolation model. Semi variance was calculated using the equation below, as follows:

(1) $$\gamma \left(h\right)=\frac{1}{2|N\left(h\right)}+\sum_{N\left(h\right)}\left(Z\left(x_{i}\right)-Z(x_{j)}\right),$$

where the number of pairs parted by distance is indicated by N(h), and Z(xi) and Z(xj) represent Z in the x_i_ and x_j_ positions, respectively.

The soil physico-chemical data were converted into shape file and kriged to identify soil characteristic of unknown location. The spatial variability maps are presented in Figs. 2, 3 and 4.

## Ethics Statement

All the authors declare that there is no ethical statement or clearance required for the presented data.

## CRediT Author Statement

**S. Sathiyamurthi:** Conceptualization, Methodology, Data analysis and manuscript reviewing and editing; **M. Ramya:** Lab analysis, data curation, and manuscript writing.

## Declaration of competing Interest

On behalf of all authors, there is no conflict of interest.

## Data Availability

raw data article on the physico-chemical properties of soil from six firkas in Dharmapuri district, Tamil Nadu, India (Original data) (Mendeley Data).
